# Thermal, Physical and Mechanical Properties of Poly(Butylene Succinate)/Kenaf Core Fibers Composites Reinforced with Esterified Lignin

**DOI:** 10.3390/polym13142359

**Published:** 2021-07-19

**Authors:** Harmaen Ahmad Saffian, Masayuki Yamaguchi, Hidayah Ariffin, Khalina Abdan, Nur Kartinee Kassim, Seng Hua Lee, Ching Hao Lee, Ayu Rafiqah Shafi, Aisyah Humairah Alias

**Affiliations:** 1 Institute of Tropical Forestry and Forest Products (INTROP), Universiti Putra Malaysia, UPM Serdang, Seri Kembangan 43400, Malaysia; hidayah@upm.edu.my (H.A.); khalina@upm.edu.my (K.A.); leechinghao@upm.edu.my (C.H.L.); ayu.rafiqah@yahoo.com (A.R.S.); aisyah.humaira@upm.edu.my (A.H.A.); 2 Japan Advance of Institute Science and Technology (JAIST), Nomi, Ishikawa 923-1292, Japan; m_yama@jaist.ac.jp; 3 Faculty of Science, Universiti Putra Malaysia, UPM Serdang, Seri Kembangan 43400, Malaysia; kartinee@upm.edu.my; 4 Faculty of Engineering, Univeristi Putra Malaysia, UPM Serdang, Seri Kembangan 43400, Malaysia

**Keywords:** poly (butylene succinate), kenaf core fibers, lignin, mechanical properties, phthalic anhydride, thermal behavior, thermal conductivity

## Abstract

In this study, Kraft lignin was esterified with phthalic anhydride and was served as reinforcing filler for poly(butylene succinate) (PBS). Composites with different ratios of PBS, lignin (L), modified lignin (ML) and kenaf core fibers (KCF) were fabricated using a compounding method. The fabricated PBS composites and its counterparts were tested for thermal, physical and mechanical properties. Weight percent gain of 4.5% after lignin modification and the FTIR spectra has confirmed the occurrence of an esterification reaction. Better thermo-mechanical properties were observed in the PBS composites reinforced with modified lignin and KCF, as higher storage modulus and loss modulus were recorded using dynamic mechanical analysis. The density of the composites fabricated ranged from 1.26 to 1.43 g/cm^3^. Water absorption of the composites with the addition of modified lignin is higher than that of composites with unmodified lignin. Pure PBS exhibited the highest tensile strength of 18.62 MPa. Incorporation of lignin and KCF into PBS resulted in different extents of reduction in tensile strength (15.78 to 18.60 MPa). However, PBS composite reinforced with modified lignin exhibited better tensile and flexural strength compared to its unmodified lignin counterpart. PBS composite reinforced with 30 wt% ML and 20 wt% KCF had the highest Izod impact, as fibers could diverge the cracking propagation of the matrix. The thermal conductivity value of the composites ranged from 0.0903 to 0.0983 W/mK, showing great potential as a heat insulator.

## 1. Introduction

Lignin is the second most abundant polymer after cellulose. Lignin originates from plants and provides mechanical support to the plants as well as acting as a binder to the fibers of plants [1]. As one of the main components in lignocellulosic biomasses, lignin is a cheap, readily available and natural substance that has huge potential to be used in bio-based material production [2]. Kraft lignin is mainly found in black liquor, a by-product generated excessively during the paper pulping process and delignification process of both softwoods and hardwoods [3]. Annually, an estimated of 1 million tons of spent pulping liquor were generated by the relevant industries all over the world. However, despite its abundancy and despite possessing high functionality, UV stabilization as well as thermostability, the utilization of Kraft lignin is still very limited. According to Kubi and Kadla [4], merely less than 2% of lignin was utilized commercially in the production of dispersants, adhesives and surfactants. Abejón et al. [5] also reported that Kraft lignin has restricted commercial applications, except for acting as a bio-based alternative in a variety of applications such as fuels, fire retardants and incorporation in various polymer blends.

Recently, in light of efforts toward stimulating the usage of lignin and expanding its commercial value, many researchers have started to use lignin as a reinforcing filler in polymeric systems [6,7,8,9]. Among the polymers, poly (butylene succinate) (PBS) is one of the vital thermoplastic polymers synthesized by polycondensation between succinic acid and butanediol [10]. It is biodegradable as it can be produced from renewable sources, making it a promising material in the composite industries [11]. However, PBS is costly compared to other conventional plastics. Therefore, incorporation of natural fibers and lignin is a common practice in order to reduce the production cost. The core fibers of kenaf (*Hibiscus cannabinus*), an annually grown crop in Malaysia, is a popular selection of reinforcements in composite panels. Incorporation of kenaf core fibers (KCF) has been reported to bestow the polymeric composites with better mechanical properties [12,13,14,15]. Nevertheless, the compatibility between hydrophobic polymers and hydrophilic natural fibers is an undesired problem that could affect the properties of the composite negatively, such as poor dimensional stability and poor durability against biological agents.

Furthermore, lignin addition could lead to decrement in tensile strength and the elongation properties of the polymeric composite [16]. This is due to the poor compatibility between the lipophilic matrix and unmodified lignin. Therefore, a direct blend of unmodified lignin with plastics is not feasible [17]. On this account, lignin commonly undergoes modification to alter its physical properties, increasing lipophilicity and reducing glass transition temperature before being blended with plastics. According to Thielemans and Wool [18], the organic solubility, thermoplasticity, and hydrophobicity of lignin could be enhanced by esterification of its hydroxyl groups. Using hydroxyl esterification, the processability of lignin could be improved as a result of reduction in hydrogen bonding and increased chain flexibility. Yue et al. [19] used aliphatic chlorides as esterification reagents to introduce aliphatic chains into the macromolecule of kraft lignin. The esterified lignin was then used as coupling agent in PBS/chemi-thermomechanical pulp fiber composites. Improved interfacial bonding between fibers and matrix was observed, accompanied by improvement in mechanical performance, water absorption and storage modulus (E) compared to that of unmodified lignin. Other chemicals such as phthalic anhydride and oleic acid could also be used as esterification reagents to modify lignin [2].

Previous studies showed that cyclic anhydrides such as maleic, succinic, and phthalic anhydride have been used in modifying lignin, and promising results were reported. Generally, better intermolecular interactions between modified lignin and polymer matrixes, as well as better thermal stability, were reported [7,20,21,22,23]. However, study on the effects of reinforcing esterified lignin into PBS/KCF composites is very limited. Among the cyclic anhydrides, phthalic anhydride was reported to have the highest reactivity [20]. Therefore, in this study, lignin was esterified by phthalic anhydride and was later reinforced into PBS/KCF composite. The effects of esterified lignin incorporation on the physical properties, mechanical strength, and thermal conductivity of the resultant composite was evaluated.

## 2. Materials and Methods

Pure lignin (alkali low sulfonate content; CAS Number: 8068-05-1) was purchased from Evergreen Engineering & Resources, Semenyih, Selangor, Malaysia. The lignin (Sigma-Aldrich, St. Louis, MI, USA) has a pH of 10.5 and a particle size of 44 ± 15 μm. Phthalic anhydride (Sigma-Aldrich, St. Louis, MI, USA) in powder form was also purchased from Evergreen Engineering & Resources, Semenyih, Selangor, Malaysia. Poly(butylene succinate) branded Sigma-Aldrich (average Mw 10,000; CAS Number 25569-53-3) and kenaf core with a fiber size of about 0.05–1.00 mm were purchased from Polycomposites Sdn.Bhd., Seremban, Negeri Sembilan, Malaysia.

### 2.1. Esterification with Phthalic Anhydride Modification

The Kraft lignin sample was oven dried at 80 °C until it reached a constant weight. The oven dried lignin was then treated with phthalic anhydride (PA). The esterification reactions were carried out as follows: the lignin was immersed in a 10% solution of organic anhydride in acetone, with a ratio of 1:20 (*w*/*v*). Afterwards, it was heated at a reflux temperature of 60 ± 2 °C for 7 h in a reactor vessel provided with an agitator. At the end of the reaction, the reaction mixture was evaporated under vacuum to remove most of the acetone. Phthalic anhydride were washed with toluene to remove unreacted anhydrides and by products, followed by filtering through a pre-weighted crucible. Furthermore, the modified lignin was washed successively with distilled water until a neutral pH was reached. The products were then vacuum oven dried at 80 °C until they reached a constant weight.

### 2.2. Preparation and Formulation of Modified Lignin-Based Composites

Table 1 shows the lignin-based composite formulation and fabrication. The mixtures of lignin (L), modified lignin (ML), poly(butylene succinate) (PBS), kenaf core fiber (KCF) and polymeric diphenylmethane diisocyanate (PMDI) were then processed in an internal mixer machine (Brabender, Italy) at a 125 °C barrel temperature, 50 rpm screw rotation (co-rotation configuration) and were compounded for 12 min. After compounding, the formulations were pressed in a hot press machine (hot and cold plate) at 130 °C for 10 min. After pressing, the samples, having dimensions of 150 mm length × 150 mm width × 2 mm thickness, were cut manually using a cutter for determination of physical, mechanical and thermal properties evaluation.

### 2.3. Characterization of Surface Lignin Modification

#### 2.3.1. Weight Percent Gain (WPG)

The extent of the reaction will be calculated as weight percent gain (WPG) determined by the differences in oven dry weight of the sample before (W_1_) and after (W_2_) modification according to the following equation:

WPG (%) = (W_2_ − W_1_)/W_1_ × 100 (1)

Five replicates were used to determine the WPG value of lignin after modification.

#### 2.3.2. Fourier Transforms Infrared Spectroscopy (FTIR)

The PBS and lignin-based composites were analyzed under the air atmosphere by using an FTIR spectrometer (Nicolet™ iS™ 10, Thermo Fisher Scientific, Inc., Waltham, MA, USA) equipped with an attenuated total reflectance accessory, without preparing KBr pellets, over the 4000–400 cm^−1^ range with a resolution of 2 cm^−1^ and 32 scans per sample. The background spectrum in the absence of any sample will be subtracted from the spectra of the individual samples. Using OriginPro 8.5 software (OriginLab Corporation, Northampton, Massachusetts, USA), baseline correction was conducted on the spectra by subtracting the baseline data. After baseline correction, the spectra were normalized into the range [1] using OriginPro 8.5 software.

#### 2.3.3. Dynamic Mechanical Analysis (DMA)

Dynamic mechanical analysis (DMA) of PBS and PBS/ML/KCF composites was carried out by a TA Q800 instrument. The samples with dimensions of 50 × 10 × 2 mm were used in the single cantilever mode. DMA scans were recorded at an oscillation frequency of 1 Hz in the temperature range from −40 to +120 °C. A heating rate of 3 °C/min was used in each experiment under a nitrogen gas flow rate of 50 mL/min.

#### 2.3.4. Thermal Conductivity Test

The thermal conductivity of all the above samples were measured by the heat flow meter apparatus (RK-30) following ASTM-C 518: “standard test method for steady state thermal transmission properties by means of the heat flow meter apparatus” and ISO 8301: “thermal insulation, determination of steady-state thermal resistance and related properties”. The thermal conductivity can be calculated from the temperatures of hot plate, cold plate, the flowing heat from hot plate to cold plate, and the thickness of the sample. The measurements were made with five replicates.

#### 2.3.5. Physical Properties

Physical properties such as density and water absorption were evaluated based on the procedures specified in ASTM D570.

##### Water Absorption Test

Sample sheets of rectangular shape with dimensions of 15 mm × 15 mm × 0.5 mm were prepared. Samples were immersed in distilled water at room temperature. After taking out samples from water, the samples were wiped dry thoroughly to remove excess amounts of water on the surfaces. The sample was weighed at two day intervals along a period of 14 days. Five measurements were performed for each sample, and the result was reported as an average value. Water absorption (WA) was calculated by Equation (2):

WA (%) = (W_2_ − W_1_)/W_2_ × 100 (2)

where, W_1_ and W_2_ are the weight of samples before and after immersion

##### Density Determination of Material and Composite Sheet

Density was determined according to the ASTM D 1895-17 (2017) standard. Five replicates were used for density determination. The density of the sample was calculated by using the following equation:

Density (g/cm^3^) = *m*/*v* (3)

where, *m* represents mass and *v* for volume.

#### 2.3.6. Mechanical Properties

##### Tensile and Flexural Test

For mechanical properties testing, a 2-mm thick plate produced through a hot-press machine was cut into a specimen shape according to ASTM D638 (tensile test) and ASTM D790 (flexural test). A mold with specific dimensions of 150 × 150 × 2 mm (length × width × thickness) was used for the sample preparation. Tensile and flexural tests were performed using a Universal Testing Machine (UTM, Instron-3366) at a crosshead speed of 5 mm/min. For every set of formulations, 5 specimens were tested to determine the average properties. Prior to testing, the specimens were conditioned at room temperature in a desiccator for 24 h.

##### Impact Test

The notched Izod impact strength was measured with a TMI Monitor impact tester (model no. 43-02-01) according to ASTM D 256, with a 5 ft lb pendulum. Notched samples, having dimensions of 63 × 12.7 × 10 (length × width × thickness), were prepared according to the standard. The notch dimensions of the samples are 2.54 mm in depth, with an angle of 45° and a tip curvature radius of 0.25. Five replicates were prepared and tested for every formulation.

#### 2.3.7. Morphological Analysis

Morphology of the samples was observed using a Hitachi S-3400N scanning electron microscope (SEM, UPM, Serdang, Malaysia) equipped with an energy dispersive X-ray (EDX, UPM, Serdang, Malaysia) under an accelerating voltage of 15 kV. The samples were gold sputtered before observation to avoid the charging effect.

## 3. Results & Discussion

### 3.1. Weight Percent Gain (WPG)

According to Abdul Khalil et al. [24], the extent of modification could be assessed by WPG value. The WPG of the lignin after esterification with phthalic anhydride in this study was 4.5 ± 0.06%. The value is very close to the value reported by Chen et al. [20], which is 5.3%. In that study, kraft lignin modified by phthalic anhydride had the highest WPG value (5.3%) compared to that of kraft lignin modified with maleic anhydride (2.9%) and succinic anhydride (4.4%). The findings indicated that phthalic anhydride has higher reactivity than the other two anhydrides.

### 3.2. Fourier Transforms Infrared Spectroscopy (FTIR)

**Figure 1:**
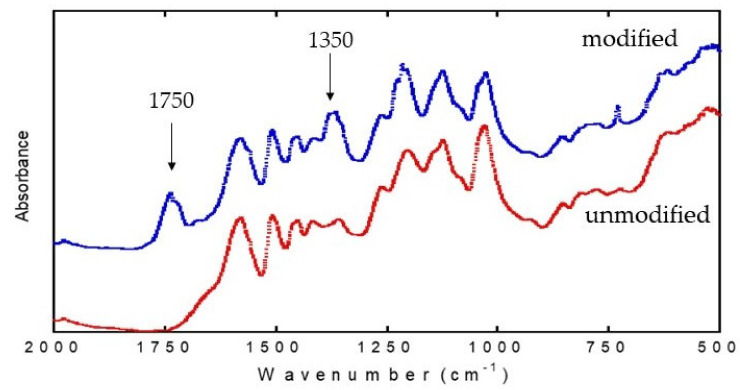
ATR-Fourier-transform infrared spectrometer (FTIR) of unmodified (red) and esterified lignin (blue).

In order to verify the esterification reaction, the normalized FTIR-ATR spectra of unmodified lignin and esterified lignin are shown in Figure 1. From the figure, the esterification reaction was confirmed by the appearance of a new peak at around 1750 cm^−1^. The peak corresponds to the carbonyl stretch (C=O) of aliphatic esters. No peak was observed in the unmodified lignin. The occurrence of this peak indicates that the esterification reaction has taken place. Apart from that, intensification of the absorption band at around 1350 cm^−1^, which corresponds to the C–O stretch of anhydride, was observed [25]. The alteration of spectra suggested that the esterification reaction has occurred, and therefore that the lignin is successfully modified by phthalic anhydride. Moreover, the results of differential scanning calorimetry (not shown in this paper) recorded a decrement in *T*_g_ of phthalic anhydride modified lignin, implying that the decrement in *T*_g_ is mainly caused by gains in the free volume of molecules as the ester substitution increases [20]. The *T*_g_ depression might also be caused by elimination of hydrogen bonds by ester substituent, as they substituted the hydroxyl groups in lignin and resulted in greater mobility within lignin molecules.

**Figure 2:**
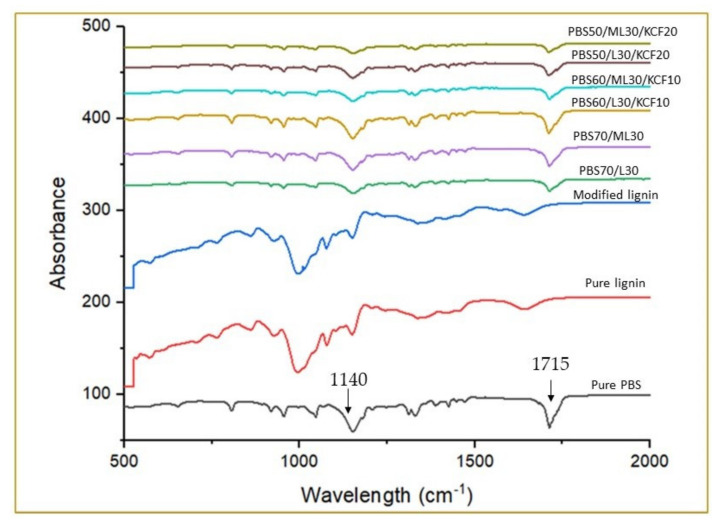
ATR-Fourier-transform infrared spectrometer (FTIR) of PBS and its composites. Note: Poly (butylene succinate) (PBS), Lignin (L), Modified Lignin (ML), Kenaf Core Fiber (KCF).

Figure 2 displays all of the normalized infrared spectra of pure PBS, unmodified lignin, modified lignin and composites made from PBS, KCF and lignin or modified lignin. Typical peaks of PBS were found near 1715 and 1140 cm^−1^ which were attributed to the C = O stretching, and C–O–C stretching of the PBS [7]. Meanwhile, all of the PBS/lignin, PBS/modified lignin, PBS/lignin/KCF and PBS/modified lignin/KCF composites had more or less similar spectra with that of pure PBS, with the exception of different intensities at the characteristic peaks.

### 3.3. Dynamic Mechanical Analysis (DMA)

**Figure 3:**
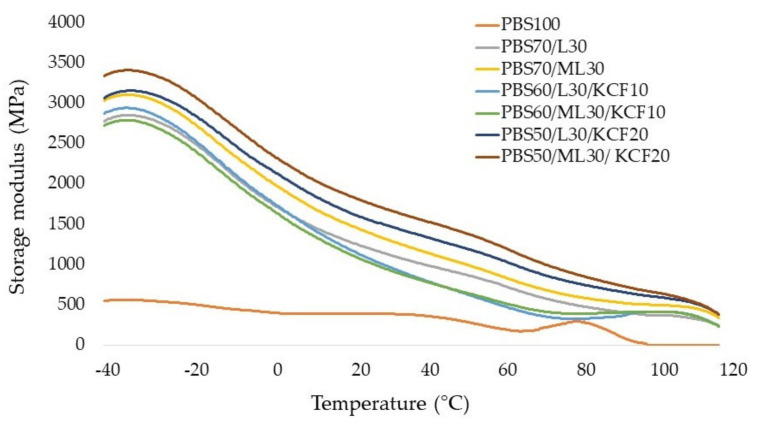
Storage modulus (E’) of the composites prepared from unmodified and modified lignin blend with PBS and kenaf core fibers.

Figure 3 exhibits the storage modulus (E’) of the PBS composites and its composites blended with unmodified or modified lignin and KCF. The storage modulus decreased along with increasing temperature, due to polymer softening as a result of increased chain mobility of the polymer matrix. The storage modulus of the PBS composites reinforced with lignin and KCF are much higher than pure PBS. PBS composites reinforced with modified lignin exhibited a higher storage modulus than that of the PBS composites with unmodified lignin. Samples PBS50/ML30/KCF20 had the highest storage modulus among all PBS composites produced in this study. Better performance of modified PBS could be attributed to the improved adhesion between fibers and the matrix. Besides, the findings also indicate that the KCF acts as a reinforcing agent for the PBS matrix, thereby increasing the stiffness of the matrix. However, such effects could only be observed when 20 wt% KCF was added. PBS composites reinforced with 10 wt% KCF displayed an inferior storage modulus than PBS70/L30 and PBS30/ML30 composites.

**Figure 4:**
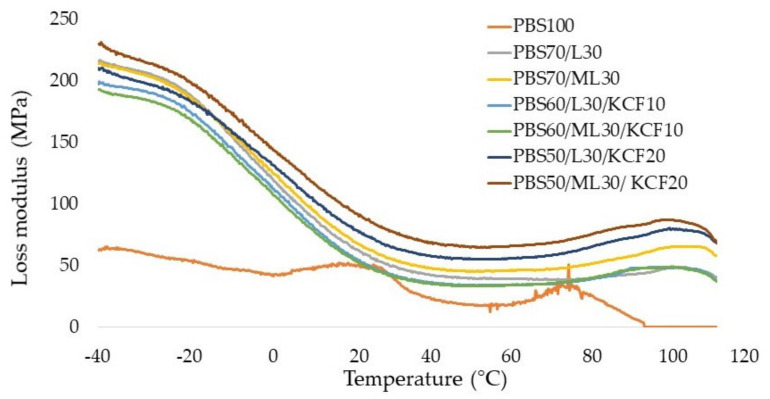
Loss modulus (E”) of the composites prepared from an unmodified and modified lignin blend with PBS and kenaf core fibers.

**Figure 5:**
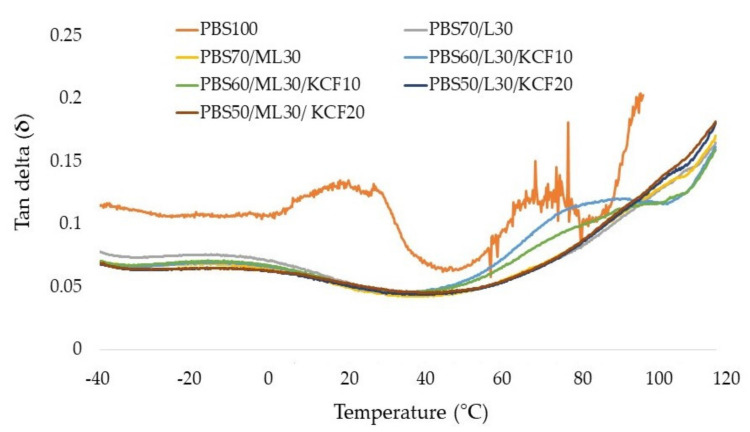
Tan delta (**δ**) of the composites prepared from an unmodified and modified lignin blend with PBS and kenaf core fibers.

Figure 4 displayed the loss modulus (E”) of the PBS composites fabricated in this study. Basically, the loss modulus trend mirrored that of the trend of storage modulus in Figure 3. At room temperature (25 °C), the loss modulus value of PBS composites in descending order is as follow: PBS50/ML30/KCF20 > PBS50/L30/KCF20 > PBS70/ML30 > PBS70/L30 > PBS60/L30/KCF10 > PBS60/ML30/KCF10 > PBS100. Two small peaks were observed from the PBS composites reinforced with KCF and lignin in Figure 4. The peak might correspond to the glass transition temperature (*T*_g_) of the polymeric phase, while the second peak is probably caused by the KCF and lignin [26]. The damping behavior, or tan δ, of the PBS composites are shown in Figure 5. The broadening of the tan δ thermograms could be observed due to the incorporation of lignin. The tan δ peak temperature (often referred as glass transition temperature, *T*_g_) decreased at the addition of lignin and KCF. Both PBS50/ML30/KCF20 and PBS50/L30/KCF20 composites showed the lowest *T*_g_, implying decrement in the molecular mobility of the composite materials, and the mechanical loss which occurred to overcome the inter-friction between molecular chains was also reduced [26]. Further, 20 wt% KCF represents a huge volume being added to the PBS composites, and therefore further restricts the molecular mobility of the composites. Therefore, PBS composites with higher loading of KCF have lower damping properties [27]. However, despite the slight decrement, there is no significant evidence of *T*_g_ shifting upon insertion of kenaf and/or lignin fillers in the PBS composites. The *T*_g_ values are also dependent on its tacticity. The gradually dropping of the storage modulus of PBS composites shows that its rubbery regime of PBS is significantly longer.

### 3.4. Physical Properties

#### 3.4.1. Density

Table 2 summarizes the density of pure PBS and its lignin based composites. Pure PBS has the lowest density of 1.26 g/cm^3^ among all of the composites produced in this study. After the addition of lignin and KCF, all of the composites displayed a higher density compared to pure PBS, where the highest density of 1.43 g/cm^3^ was recorded in the PBS70/L30 sample.

#### 3.4.2. Water Absorption

**Figure 6:**
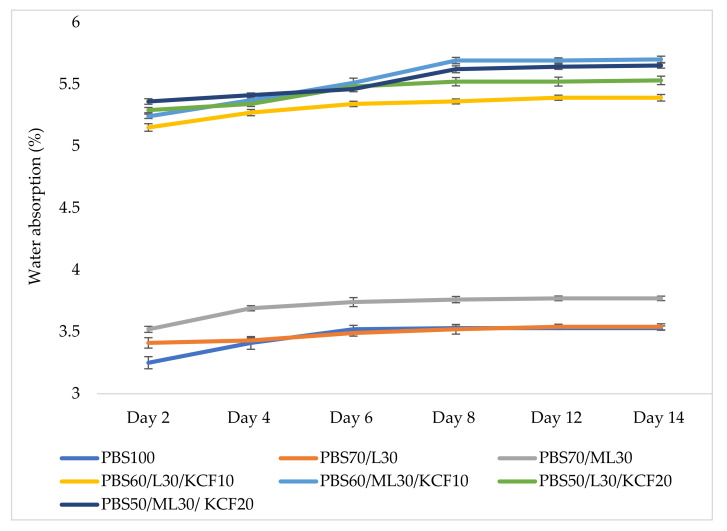
Water absorption of PBS and its composites. Note: Poly (butylene succinate) (PBS), Lignin (L), Modified Lignin (ML), Kenaf Core Fiber (KCF).

The water absorption behavior of PBS and its lignin based composites over a soaking period of 14 days are displayed in Figure 6. From Figure 6, one can see that pure PBS has the lowest water absorption of 3.25% after two days immersion, and the water absorption became stagnant after eight days and is maintained at 3.53% until day 14. After the addition of unmodified lignin and modified lignin, the water absorption of the composites increased slightly to 3.41% and 3.52%, respectively, after two days immersion. At day 14, water absorption values of 3.54% and 3.77% were recorded, respectively. However, the water absorption of the composites increased markedly after 10% and 20% KCF was added. The water absorption of all of the composite samples containing KCF ranged from 5.39% to 5.70% after 14 days of water immersion. This is an expected observation due to the inherent hydrophilic behavior of KCF. In addition, the formation of voids as a result of structural inhomogeneity between KCF and PBS has also contributed to the increased water absorption of the composites [28].

Meanwhile, when comparing unmodified and modified lignin, addition of modified lignin slightly increased the water absorption rate of the resultant composites. Based on Figure 6, the absorption rate of the composites with addition of modified lignin is higher than that of composites with unmodified lignin. The trend becomes more obvious after day eight, where both PBS60/ML30/KCF10 and PBS50/ML30/KCF20 exhibited the highest water absorption compared to the other counterparts. The phenomena may be due to the poor interfacial bonding that developed micro-cracks and voids in the composites [29]. Many factors such as porosity, void content, lumen size, and fiber-matrix adhesion are able to affect the water absorption behavior of the composites [30]. The inter-fibril spaces of cellulosic structures of natural fibers allow the retention of water molecules [31]. Apart from this, hydroxyl groups on the lignin constituents could also contribute to the increased water absorption [32]. It is known that the hydroxyl groups have a strong tendency to form hydrogen bonds with water molecules [33]. Modified lignin exhibited a rougher surface, as confirmed by the SEM images at the latter part of this paper. Therefore, it might result in higher water retention on the surface of modified lignin and lead to higher water absorption.

### 3.5. Mechanical Properties

**Figure 7:**
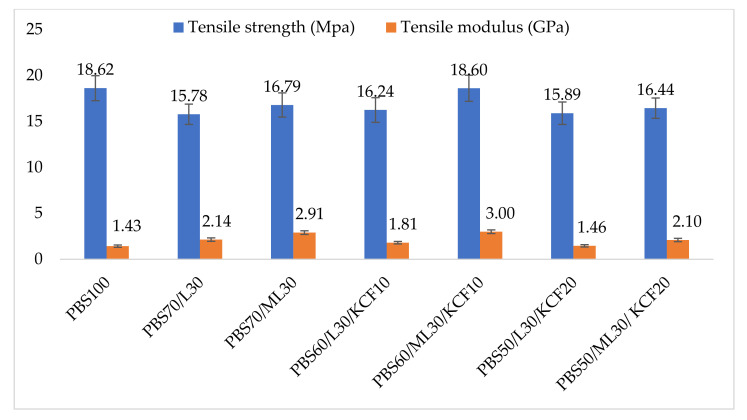
Tensile properties of PBS and its composites. Note: Poly (butylene succinate) (PBS), Lignin (L), Modified Lignin (ML), Kenaf Core Fiber (KCF).

The tensile properties of the pure PBS composite and its lignin based composites are displayed in Figure 7. Based on the observation from Figure 7, pure PBS exhibited the highest tensile strength of 18.62 MPa. Incorporation of lignin and KCF into PBS resulted in a different extent of reduction in tensile strength (15.78 to 18.60 MPa). After being reinforced with 30 wt% unmodified and modified lignin, the tensile strength of the PBS composites reduced. However, tensile strength of the composite increased when 10 wt% KCF was added and decreased again when the KCF loading reached 20 wt%. Sahoo et al. [26] attributed the reduction in tensile strength to the poor interfacial adhesion between filler and matrix. Generally, composites with modified lignin exhibited better tensile strength than its unmodified lignin counterparts. As the modification by phthalic anhydride has removed most of the hydrophilic hydrogen bonding of the lignin, a better interfacial adhesion is therefore achieved. The improved interfacial adhesion between PBS and modified lignin has a positive impact on the stress transfer, by reducing the chance of interfacial debonding.

However, the tensile modulus of the composites behaves differently compared to tensile strength. The tensile modulus of pure PBS is 1.43 GPa, and increased to 1.46–3.00 GPa when lignin and KCF were added. The findings were in agreement with Ahmad Saffian et al. [7], who reported an increase in the tensile modulus after malleated lignin was reinforced into PBS polymer. The improvement in the properties of composites indicates an interaction, possibly a polar–polar interaction between lignin and the PBS matrix. The interaction was due to the presence of a polar carbonyl group [34]. Therefore, a hydrogen bond formation could be possible between the carbonyl group of the PBS matrix and the hydroxyl group of lignin.

**Figure 8:**
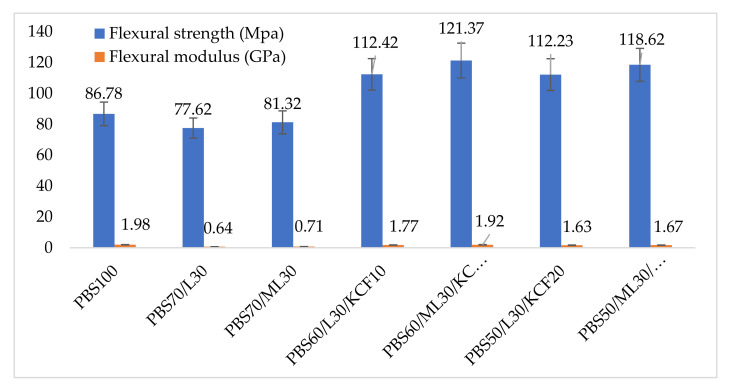
Flexural properties of PBS and its composites. Note: Poly (butylene succinate) (PBS), Lignin (L), Modified Lignin (ML), Kenaf Core Fiber (KCF).

Figure 8 shows the flexural properties of the PBS and its lignin based composites. Pure PBS has a flexural strength of 86.78 MPa. A 10.6% and 6.29% reduction in flexural strength was observed when 30 wt% unmodified and modified lignin was added, respectively. Nevertheless, the flexural strength of the composites increased (112.23 to 121.37 MPa) when 10 wt% and 20 wt% KCF was reinforced into the composites. A similar trend was also observed for the flexural modulus of the composites. Flexural modulus of 1.98 GPa was recorded in pure PBS, and it decreased when lignin was incorporated. However, when KCF was added, the flexural modulus increased and ranged between 1.63 to 1.92 GPa. Greater stress transfer from the matrix to fiber through a modified lignin interface could be attributed to this significant improvement.

**Figure 9:**
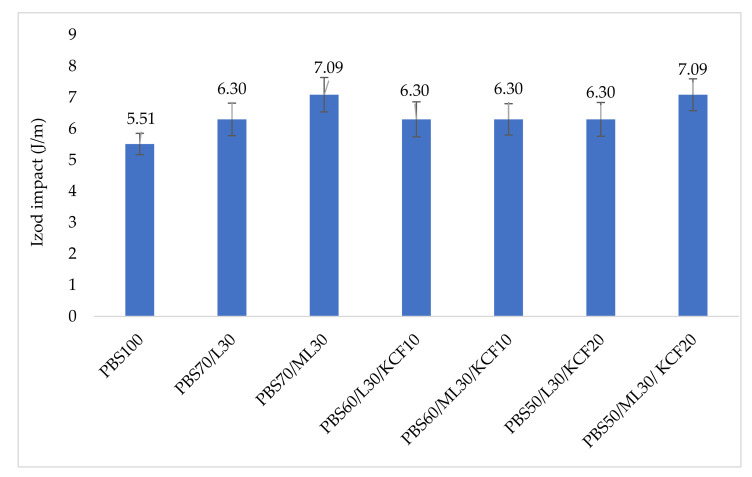
Impact test for PBS and its composites. Note: Poly (butylene succinate) (PBS), Lignin (L), Modified Lignin (ML), Kenaf Core Fiber (KCF).

The Izod impact test determines the energy that a material can absorb through the help of a standardized high strain rate test. The energy absorbed is used to determine the toughness of the material and how resistant it is to the impact. The Izod impact of the PBS and its lignin based composites are illustrated in Figure 9. Pure PBS has an Izod impact of 5.51 J/m. Composites with addition of lignin and KCF have a better Izod impact than pure PBS. The highest Izod impact of 7.09 J/m was recorded in PBS70/L30 and PB50/ML30/KCF20. Reinforcement with KCF or any natural fiber increased the ductility of the resultant composites. Costa et al. [35] reported the role of natural fibers in the fracture mechanism, and concluded that the existence of natural fibers allowed the absorption of higher impact energy. Dissimilar to the brittle nature of pure PBS, natural fibers in composites can act as effective barriers that diverge the cracking propagation of the matrix. Consequently, higher absorbed impact energy could be observed in those composites reinforced with natural fibers. The same explanation could also be applied to composites reinforced with lignin (PBS70/L30 & PBS70/ML30), as small lignin particles could block crack propagation and lead to better impact strength. Apart from this, impact strength of a composite is also dependent on the dispersion of the filler particles [36]. Modified lignin resulted in a better impact strength than unmodified, which could be explained by the fact that modified lignin could be better dispersed into the PBS matrix.

### 3.6. Thermal Conductivity

The thermal conductivity of the PBS/KCF composites reinforced with unmodified and esterified lignin are shown in Table 3. The thermal conductivity value of the composites ranged from 0.0903 to 0.0983 W/mK. It was observed that the addition of lignin and KCF slightly increased the thermal conductivity of the PBS/KCF composites. Regardless of the fiber loading of KCF, all of the composites reinforced with esterified lignin showed a slight increment in thermal conductivity. Asdrubali et al. [37] stated that a thermal insulator should possess a thermal conductivity value of 0.07 W/mK and lower. However, the thermal conductivity of all composites produced in this study is slightly higher than the aforementioned value. On the other hand, Karwa et al. [38] claimed that materials having a thermal conductivity of 0.25 W/mK and below could be used as a heat insulator. Therefore, the PBS/KCF composites in this study showed great potential as heat insulators.

### 3.7. Morphology Properties

**Figure 10:**
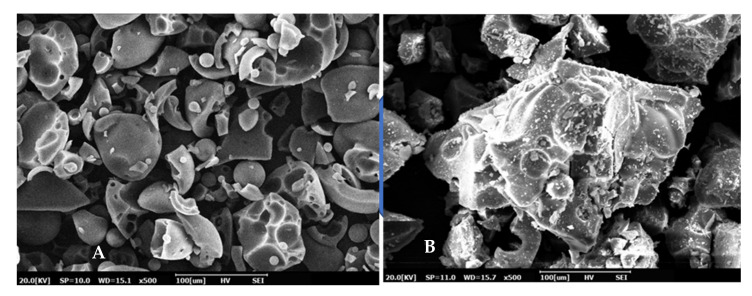
Morphology of pure lignin (**A**) before modification, and lignin after modification by phthalic anhydride (**B**).

**Figure 11:**
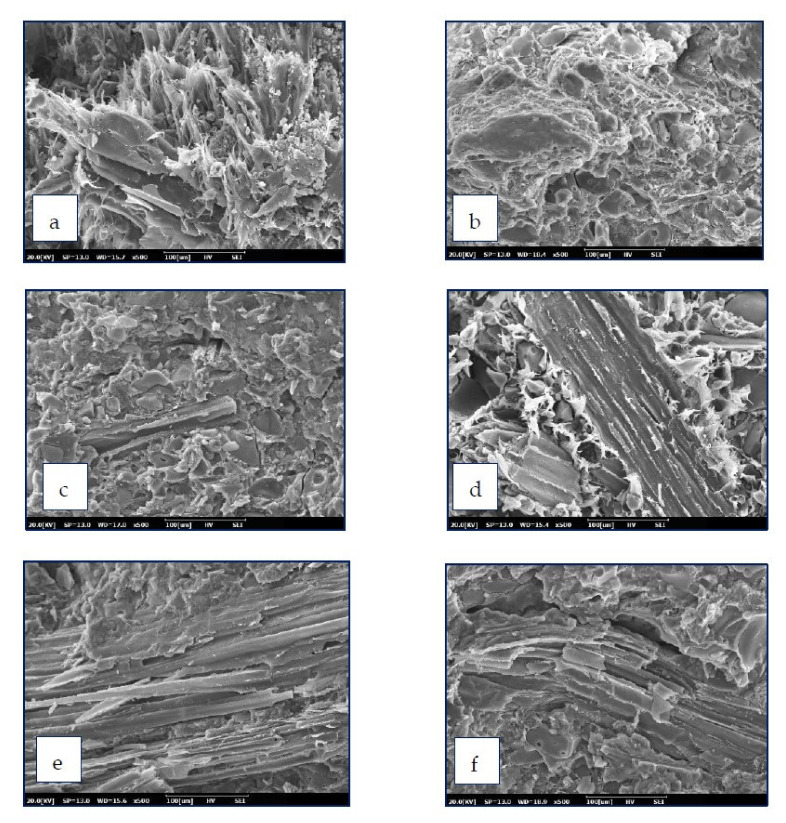
SEM micrograph of PBS composites: (**a**) PBS70/L30, (**b**) PBS70/ML30, (**c**) PBS60/L30/KCF10, (**d**) PBS60/ML30/KCF10, (**e**) PBS50/L30/KCF20, and (**f**) PBS50/ML30/KCF20.

The morphology of the lignin before and after modification are shown in Figure 10. Pure lignin exhibited an irregular block structure and a smooth surface. It can be observed that lignin was covered by phthalic anhydride after modification. The surface of the modified lignin became rougher, mainly due to the removal of hydroxyl groups and elimination of intermolecular hydrogen bonding by esterification reactions [39]. This finding explained the reason why *T*_g_ of composites prepared by PBS/ML and PBS/ML/KCF were lower than that of unmodified samples mentioned in the above section. The SEM photographs of the PBS composites are shown in Figure 11. PBS composites with the addition of KCF (Figure 11c–f) had rougher surfaces compared to that of PBS composites with solely lignin (Figure 11a,b). Tensile data from previous sections suggests that addition of KCF improved the tensile strength of the PBS composites compared to that of PBS composites reinforced with only lignin. It was reported that KCF could provide greater stress transfer and diverge crack propagation. Therefore, PBS composites with the addition of KCF displayed higher tensile strength.

## 4. Conclusions

In this study, esterified lignin and kenaf core fibers were used as reinforcement filler for poly(butylene succinate) (PBS) composites. Overall, better storage modulus was recorded in the PBS/KCF composite reinforced with esterified lignin compared to that of unmodified lignin. However, PBS composites reinforced with KCF and lignin displayed lower damping properties. In terms of physical properties, composites blended with modified lignin and KCF exhibited higher water absorption even after soaking for two days, and continued to increase until day 14. As for tensile strength, composites fabricated from modified lignin recorded higher tensile strength and tensile modulus compared to unmodified samples. A similar trend was also observed for flexural strength and flexural modulus, where PBS/KCF composites reinforced with modified lignin showed higher values than their unmodified counterparts. The improvement in mechanical properties was mainly due to better interfacial bonding between the filler and the matrix. The thermal conductivity study suggested that PBS/lignin/KCF composites have the potential to be used as heat insulators.

## Figures and Tables

**Table 1:** PBS composites formulation and fabrication.

Comosites	Code	PBS (%)	Lignin (%)	PMDI (%)	KCF (%)
PBS	PBS100	100	0	3	0
PBS/Lignin	PBS70/L30	70	30	3	0
PBS/Modified Lignin	PBS70/ML30	70	30	3	0
PBS/Lignin/KCF	PBS60/L30/KCF10	60	30	3	10
PBS/Modified Lignin/KCF	PBS60/ML30/KCF10	60	30	3	10
PBS/Lignin/KCF	PBS50/L30/KCF20	50	30	3	20
PBS/Modified Lignin/KCF	PBS50/ML30/KCF20	50	30	3	20

**Table 2:** Density of PBS and its composites.

Composites	Density (g/cm^3^)
PBS100	1.26 ± 0.26
PBS70/L30	1.43 ± 0.21
PBS70/ML30	1.41 ± 0.08
PBS60/L30/KCF10	1.37 ± 0.46
PBS60/ML30/KCF10	1.39 ± 0.78
PBS50/L30/KCF20	1.34 ± 0.72
PBS50/ML30/KCF20	1.38 ± 0.68

Note: Poly (butylene succinate) (PBS), Lignin (L), Modified Lignin (ML), Kenaf Core Fiber (KCF).

**Table 3:** Thermal conductivity and its composites.

Composites	Thermal Conductivity (W/mk)
PBS100	0.0903 ± 0.0005
PBS70/L30	0.0942 ± 0.0009
PBS70/ML30	0.0970 ± 0.0013
PBS60/L30/KCF10	0.0918 ± 0.0009
PBS60/ML30/KCF10	0.0983 ± 0.0005
PBS50/L30/KCF20	0.0965 ± 0.0007
PBS50/ML30/KCF20	0.0983 ± 0.0005

## Dedication

In loving memory of Harmaen Ahmad Saffian, a passionate researcher whose light we lost far too soon.
